# Prevalence of treatment resistance and clozapine use in early intervention services

**DOI:** 10.1192/bjo.2020.89

**Published:** 2020-09-07

**Authors:** Imogen Stokes, Siân Lowri Griffiths, Rowena Jones, Linda Everard, Peter B. Jones, David Fowler, Joanne Hodgekins, Tim Amos, Nick Freemantle, Vimal Sharma, Max Marshall, Swaran P. Singh, Max Birchwood, Rachel Upthegrove

**Affiliations:** Birmingham Medical School, College of Medical and Dental Sciences, University of Birmingham, UK; School of Psychology, Institute for Mental Health, University of Birmingham, UK; School of Psychology, Institute for Mental Health, University of Birmingham; and Research and Innovation, Birmingham and Solihull Mental Health Foundation Trust, UK; Research and Innovation, Birmingham and Solihull Mental Health Foundation Trust, UK; University of Cambridge, UK; Department of Psychology, University of Sussex, UK; Norwich Medical School, University of East Anglia, UK; University of Bristol, UK; Institute of Clinical Trials & Methodology, University College London, UK; Faculty of Health and Social Care, University of Chester, UK; Lancashire Care NHS Foundation Trust, UK; Birmingham Early Intervention Service, Birmingham Women's and Children's NHS Trust, UK; University of Warwick, UK; Birmingham Medical School, College of Medical and Dental Sciences, University of Birmingham; School of Psychology, Institute for Mental Health, University of Birmingham; Birmingham Early Intervention Service, Birmingham Women's and Children's NHS Trust, UK

**Keywords:** Treatment resistance, schizophrenia, clozapine, early psychosis, early intervention

## Abstract

**Background:**

Treatment resistance causes significant burden in psychosis. Clozapine is the only evidence-based pharmacologic intervention available for people with treatment-resistant schizophrenia; current guidelines recommend commencement after two unsuccessful trials of standard antipsychotics.

**Aims:**

This paper aims to explore the prevalence of treatment resistance and pathways to commencement of clozapine in UK early intervention in psychosis (EIP) services.

**Method:**

Data were taken from the National Evaluation of the Development and Impact of Early Intervention Services study (*N* = 1027) and included demographics, medication history and psychosis symptoms measured by the Positive and Negative Syndrome Scale (PANSS) at baseline, 6 months and 12 months. Prescribing patterns and pathways to clozapine were examined. We adopted a strict criterion for treatment resistance, defined as persistent elevated positive symptoms (a PANSS positive score ≥16, equating to at least two items of at least moderate severity), across three time points.

**Results:**

A total of 143 (18.1%) participants met the definition of treatment resistance of having continuous positive symptoms over 12 months, despite treatment in EIP services. Sixty-one (7.7%) participants were treatment resistant and eligible for clozapine, having had two trials of standard antipsychotics; however, only 25 (2.4%) were prescribed clozapine over the 12-month study period. Treatment-resistant participants were more likely to be prescribed additional antipsychotic medication and polypharmacy, instead of clozapine.

**Conclusions:**

Prevalent treatment resistance was observed in UK EIP services, but prescription of polypharmacy was much more common than clozapine. Significant delays in the commencement of clozapine may reflect a missed opportunity to promote recovery in this critical period.

Psychosis is a common, often disabling disorder that occurs at a critical time in a young person's development. Despite advances in mental health treatment, the outcomes for psychosis remain poor for many.^1^ A recent meta-analytic review of longitudinal outcomes in first-episode psychosis (FEP) reported a 38% pooled recovery rate.^2^ Other systematic reviews have explored relapse and recovery rates following medication discontinuation in FEP; although there is a variation in the rates reported across studies (19–89%), the risk of relapse is significantly reduced by sustained antipsychotic therapy.^3–5^ These findings have important consequences for the selection of interventions in FEP.^5^

Birchwood and colleagues proposed the concept of a ‘critical period’ in the development and treatment of psychosis,^6–8^ with sustained and intensive intervention within early intervention in psychosis (EIP) services potentially improving outcomes. Adopting an assertive outreach community framework, EIP services within the UK offer a range of treatment modalities in addition to psychopharmacology, including psychosocial, vocational and family interventions to promote recovery.^6,9^

Such intensive early treatment includes the identification and active management of early treatment-resistant symptoms. In England, EIP services are now highly developed and monitored for the identification of such treatment resistance, which can be defined as the continued presence of symptoms despite the adequate trial of two antipsychotic medications, and the offer of clozapine to individuals who meet these criteria.^10,11^

## Management of treatment resistant psychosis

Although the response rate to antipsychotic medication in the early phase of psychosis is generally good compared with established cases,^12^ clozapine is the only available medication with proven efficacy for patients with treatment-resistant schizophrenia.^13,14^ Clozapine has superior efficacy in reducing symptom burden and suicide, and in improving functioning in patients with treatment-resistant psychosis.^15^ It is also shown to substantially reduce mortality rates in individuals with schizophrenia.^10^ Demjaha et al brought to light the large proportion of patients who were treatment resistant from the outset of their FEP, and recommended clozapine treatment as early as possible during the first presentation of psychosis.^16^ However, literature suggests that clinicians are more inclined to prescribe a higher dose of a standard antipsychotic than recommended, rather than prescribe clozapine.^10,15^ Furthermore, patients eligible for treatment with clozapine were found to face delays in commencement of treatment, ranging from 19.3 weeks to 5.5 years.^15^ Other literature suggests delays in utilising clozapine are even more extensive; Wheeler carried out a retrospective chart review of adult out-patients in New Zealand, finding an average duration of illness of 9.7 years before initiation of clozapine.^17^ In 2017, Doyle et al studied a cohort of patients with FEP and demonstrated that clozapine was significantly underutilised, yet after the initiation of clozapine, the mean number of hospital admissions significantly reduced.^18^

## The present study

The National Evaluation of the Development and Impact of Early Intervention Services (EDEN) study is the largest cohort study of young people with FEP, who received care under comprehensive early intervention services in the UK.^19^ This paper aims to utilise this comprehensive, longitudinal study data to present the prescribing patterns of antipsychotic medication and present the pathways to, and prescribing of, clozapine for treatment of early treatment-resistant psychosis.^11^ Furthermore, this paper aims to explore the wider prescribing patterns of psychiatrists in UK-based EIP services in the pharmacologic management of FEP.

## Method

### Study overview

Data used were from the longitudinal, seven-site UK National EDEN study. Recruitment concluded in April 2009, with the final 12-month follow-up completed by April 2010.^19^ Data for this paper included patient demographics, full medication history and Positive and Negative Syndrome Scale (PANSS) score.^20^ Pathways for those with treatment resistance both with and without clozapine, and the co-prescribing of other psychotropic mediation (e.g. antidepressants), are presented.

The authors assert that all procedures contributing to this work comply with the ethical standards of the relevant national and institutional committees on human experimentation and with the Helsinki Declaration of 1975, as revised in 2008. All procedures involving human patients were approved by Suffolk Local Research Ethics Committee, UK (approval number 05/Q0102/44). Written or verbal informed consent was obtained from all patients. Verbal consent was witnessed and formally recorded.

### Inclusion and exclusion criteria

The National EDEN studies enrolled patients with FEP (ICD-10 diagnosis codes F29, F20, F25, F31, F32.0-F32.1, F32.3 and F30.2^21^) from early intervention services across England, including Birmingham, Cornwall, Cambridge, Norwich and Lancashire. As the study progressed, four other early intervention services were added into the study to increase the diversity of demographics; these included Solihull, Cheshire and Wirral, Peterborough and Kings Lynn. The National EDEN studies included consented patients aged 14–35 years, with a first presentation of psychosis symptoms; see Birchwood et al for the full study description.^19^

### Baseline and follow-up measures

The National EDEN study recorded baseline demographics of the entire cohort (*N* = 1027), in addition to full medication record. Severity of psychosis symptoms was measured with the PANSS, which is a widely used and validated scale.^19,20^ These measures were collected at baseline, 6 months and 12 months, by trained research assistants.

Although there are clear international criteria for remission, the agreed definitions for treatment resistance in established schizophrenia require repeated episodes and functional impairment.^13,22,23^ There are no internationally agreed criteria for treatment resistance after first episode, where diagnoses are more fluid, and positive symptoms are generally more responsive.^24^ Therefore, in line with previous literature, we used strict criteria for persistent positive symptoms (‘treatment resistance’) of a PANSS positive score ≥16 (equating to at least two positive items of at least moderate severity) at all three time points, to capture those participants most likely to be unresponsive to antipsychotic medication after FEP.^16^ Those identified as having treatment resistance, and who had been treated with at least two different antipsychotic medication, were identified as eligible for clozapine.^25,26^

### Analysis

The prescribing patterns at baseline were explored descriptively to determine the overall percentage of each medication type prescribed for the full sample (*N* = 1027). A percentage breakdown of medication class (e.g. antipsychotic, antidepressant, mood stabilisers and anxiolytics) was calculated to explore comorbid prescribing within the cohort. To explore polypharmacy within the treatment-resistant patient cohort, the full prescribing history from baseline to 12 months was scrutinised; the prescribing history was examined for co-prescribing of antipsychotics and for co-prescribing of antipsychotics with an antidepressant. The duration of clozapine prescriptions was also examined within the group prescribed clozapine.

## Results

### Sample

A total of 1027 participants consented to participate in the National EDEN study, of which 75% (*n* = 791) were successfully followed up from study entry to 12-month follow-up, with high retention of data across clinical measures.^19^ The full baseline sample had a mean age of 23 years (s.d. 5.08), 69% were male and 73% were White. Based on Operational Criteria Checklist (OPCRIT) criteria,^27^ the majority of the sample (47%) had a diagnosis of schizophrenia spectrum disorder (ICD-10 diagnosis codes F29, F20 and F25).^19^ The sample characteristics for the 791 individuals followed up to 12 months were as follows: mean age 22.58 (s.d. 4.96), 68.4% male and 74.2% White (see Table 1). OPCRIT diagnoses were only assessed at baseline.

**Table 1:** Baseline sample characteristics by treatment group

	Full sample at baseline (*N* = 1027)	Non-clozapine group (*n* = 56)	Clozapine group (*n* = 25)	*P* value
Age at onset				
Mean (s.d.)	23 (5.08)	22.93 (5.01)	22.12 (4.89)	0.501
95% CI		21.59–24.27	20.10–24.14	
Gender				
Male	709 (69.0%)	40 (71.4%)	15 (60.0%)	
Female	250 (31.0%)	16 (28.6%)	10 (40.0%)	0.309
Ethnicity				
Asian	157 (15.3%)	6 (10.7%)	8 (32.0%)	
Black	71 (6.9%)	2 (3.5%)	2 (8.0%)	
Mixed	43 (4.2%)	2 (3.5%)	1 (4.0%)	0.132
White	750 (73.0%)	45 (80.4%)	14 (56.0%)	
Other	6 (0.6%)	1 (1.8%)	0 (0.0%)	
ICD-10 Diagnosis				
Unspecified psychosis	203 (19.8%)	10 (17.9%)	6 (24.0%)	
Schizophrenia	478 (46.5%)	35 (62.5%)	15 (60.0%)	
Schizoaffective	70 (6.8%)	2 (3.5%)	0 (0.0%)	
Bipolar affective disorder	19 (1.9%)	5 (8.9%)	1 (4.0%)	0.578
Mania with psychosis	25 (2.4%)	1 (1.8%)	0 (0.0%)	
Depressive disorder	91 (8.9%)	0 (0.0%)	0 (0.0%)	
Hypomania disorder	10 (1.0%)	0 (0.0%)	0 (0.0%)	
Undetermined	131 (12.8%)	3 (5.4%)	3 (12.0%)	

Ethnicity, gender and diagnoses in non-clozapine and clozapine groups, compared by chi-squared analysis, with a *t*-test performed for age.

A total of 143 participants were identified as treatment resistant by a continuously raised PANSS positive subscore total of ≥16 at baseline, 6 months and 12 months. Of these, 61 were eligible for clozapine based on having treatment-resistant symptoms and having been treated with at least two different antipsychotic medications. See Table 1 for sample characteristics of the treatment-resistant groups.

Only 25 participants had been offered a prescription of clozapine by the 12-month time point, including 9 that had been identified as treatment resistant by the defined criteria, and 16 who had been started on clozapine where treatment resistance had not been captured at the follow-up time points. A further 56 participants were identified as treatment resistant and eligible for clozapine (meeting our criteria for treatment resistance and having been treated with two or more antipsychotic medications), but were not prescribed clozapine over the 12-month period.

### Prescribing patterns

A total of 1746 individual (psychotropic) medications were prescribed across the full sample (*N* = 1027) at baseline (Table 2). There were 1157 prescriptions for antipsychotics (66.3% of all prescriptions), 334 prescriptions for antidepressants (19.1% of all prescriptions), 334 prescriptions for anxiolytics (11.9% of all prescriptions) and 47 prescriptions for mood stabilisers (2.7% of all prescriptions), 6 of which of were lithium carbonate (0.3% of all prescriptions, 12.8% of mood stabiliser prescriptions).

**Table 2:** Breakdown of all prescriptions in the study sample

Medication type	Number of prescriptions	Percentage of prescribing
Breakdown of prescriptions (*n* = 1746) from the full study sample (*N* = 1027)		
Antipsychotics	1157	66.3%
Antidepressants	334	19.1%
Anxiolytics	208	11.9%
Mood stabilisers	47	2.7%
Five most commonly prescribed antipsychotic medications (total prescriptions *n* = 1157) from the full study sample (*N* = 1027)		
Olanzapine	412	35.6%
Risperidone	339	29.3%
Aripiprazole	125	10.8%
Quetiapine	121	10.5%
Haloperidol	46	4.0%
Other	114	9.9%
Five most commonly prescribed antidepressant medications (total prescriptions *n* = 334) from the full study sample (*N* = 1027)		
Citalopram	151	45.2%
Fluoxetine	90	26.9%
Mirtazapine	37	11.1%
Sertraline	23	6.9%
Escitalopram	11	3.3%
Other	22	6.6%
Breakdown of the polypharmacy in the ‘treatment resistant’ (persistently raised PANSS positive score) group (*n* = 143)		
Co-prescribed two antipsychotics	54	37.8%
Co-prescribed three antipsychotics	9	6.3%
Co-prescribed four antipsychotics	4	2.3%
Co-prescribed an antidepressant with an antipsychotic	57	39.9%
Co-prescribed an antidepressant with two antipsychotics	15	10.4%
Co-prescribed an antidepressant with three antipsychotics	3	2.1%
Co-prescribed an antidepressant with four antipsychotics	1	0.7%

PANSS, Positive and Negative Syndrome Scale.

Analysis of all antipsychotic prescriptions (*n* = 1157) showed that the five most commonly prescribed antipsychotics were olanzapine (19.4%), risperidone (7.2%), aripiprazole (6.9%), quetiapine (2.6%) and haloperidol (1.7%). In comparison, clozapine made up only 0.3% of antipsychotic prescriptions.

Analysis of all antidepressant prescriptions (*n* = 334) showed that the five most commonly prescribed antidepressants were citalopram (45.2%), fluoxetine (26.9%), mirtazapine (11.1%), sertraline (6.9%) and escitalopram (3.3%).

### Prescribing in treatment-resistant participants

Analysis of polypharmacy in the treatment-resistant group showed that, within the 12-month follow-up window, 54 (37.8%) participants were co-prescribed two antipsychotics, 9 (6.3%) were co-prescribed three antipsychotics and 4 (2.3%) were co-prescribed four antipsychotics. Moreover, the analysis found that many participants were co-prescribed antidepressants with an antipsychotic: 57 (39.9%) participants were co-prescribed an antidepressant with a single antipsychotic, 15 (10.4%) were co-prescribed alongside two antipsychotics, 3 (2.1%) were co-prescribed alongside three antipsychotics and 1 person (0.7%) was co-prescribed an antidepressant alongside four antipsychotics**.**

With regards to medication adherence, there was a significant difference between adherence ratings of the treatment-resistant group compared with the remaining participants (not identified as treatment resistant). Less than a quarter of the treatment-resistant group (18.8%) were actively engaged with their treatment, and 9.7% refused (or partially refused) their treatment (Table 3). This is compared with 35% and 5.5%, respectively, in the remaining sample (Table 3).

**Table 3:** Average medication adherence score for the treatment-resistant group compared with the remaining study sample

	Study sample (non-treatment resistant), *n* = 871	Treatment-resistant group, *n* = 156	*χ*^2^	*P* value
Medication adherence, *n* (%)				
Complete refusal	10 (1.5%)	4 (2.8%)		
Partial refusal	31 (4.8%)	10 (6.9%)	9.7 *v.* 5.5	
Accepts only because it is compulsory	39 (6.0%)	17 (11.8%)	23.16	0.001
Occasional reluctance	51 (7.9%)	17 (11.8%)		
Passive acceptance	158 (24.4%)	30 (20.8%)		
Moderate participation	130 (20.1%)	39 (27.1%)		
Active participation	229 (35.3%)	27 (18.8%)		
Missing cases	223	12	–	–

### Pathways to clozapine

**Fig. 1:**
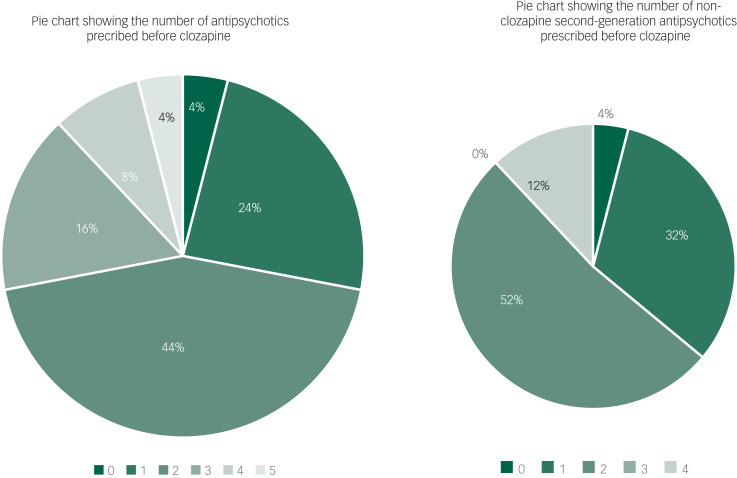
Pie charts showing the number of antipsychotics and second-generation antipsychotics prescribed before clozapine.

Participants were trialled on up to five different antipsychotics before being prescribed clozapine: 4% of patients were not trialled on an antipsychotic before being prescribed clozapine, 24% were prescribed after one antipsychotic, 44% were prescribed after two antipsychotics, 16% were prescribed after three antipsychotics, 8% were prescribed after four antipsychotics and 4% were prescribed after five antipsychotics. The mean duration of time spent on clozapine was 5.44 months, and the median duration of clozapine was 5.50 months (see Fig. 1).

Furthermore, before being prescribed clozapine, 4% of patients were not trialled on a second-generation antipsychotic, 32% were prescribed one non-clozapine second-generation antipsychotic, 52% were prescribed two different non-clozapine second-generation antipsychotics, 0% were prescribed three different non-clozapine second-generation antipsychotics and 12% were prescribed four different non-clozapine second-generation antipsychotics (see Fig. 1).

## Discussion

This data examination has described the prescribing practice and patterns to clozapine use in a large, national sample of individuals with FEP, with several findings of note. First, treatment resistance (here defined as a persistently raised PANSS positive score) is common in early intervention services, with nearly 20% of individuals having persistent high levels of symptoms despite intensive EIP care. Second, despite continuing positive symptoms, a large number of individuals remain on the same initial medication, and hence did not meet the eligibility criteria for clozapine treatment. Of those who were eligible, low numbers were prescribed clozapine. Second-generation antipsychotics were prescribed for the majority of FEP individuals, with nearly 20% of antipsychotic prescriptions at baseline being olanzapine. A total of 39.9% of participants were co-prescribed an antidepressant with an antipsychotic, and 37.8% of participants were co-prescribed at least two antipsychotics.

The rates of treatment resistance in our large sample are comparable with those found by Demjaha et al, who reported that 23% of patients experiencing FEP were treatment resistant, as defined by National Institute of Health and Care Excellence (NICE) guidelines from a sample of 323 participants experiencing FEP, studied from first contact to 10-year follow-up, from services across south-east London and Nottingham.^16^

All participants in the National EDEN study were recruited from highly concordant, specialist early interventions services, and this highlights the fact that despite intensive psychosocial interventions offered as standard in EIP services, treatment resistance does emerge,^19^ and may need specialist attention. Notably, some participants were trialled on up to five antipsychotics before being prescribed clozapine. These findings indicate a clear stasis in treatment progression, despite patients demonstrating persistent symptoms on their current regime. Two longitudinal studies have shown that of those who were identified as treatment resistant, 70% with first-episode schizophrenia and 84% with FEP were treatment resistant from illness onset, highlighting that prompt consideration of clozapine may be beneficial in this group.^28,16^

National guidelines and early intervention quality standards advocate use of clozapine for schizophrenia for illness ‘that has not improved despite the sequential use of adequate doses of at least two different antipsychotic drugs’.^25^ At least one of the drugs should be a non-clozapine second-generation antipsychotic.^26^ However, there appears to be a hesitancy to prescribe clozapine for eligible patients, with only a minority of patients in this sample prescribed clozapine after being trialled on two different antipsychotics.

It is apparent from our analysis that clinicians are continuing ineffective antipsychotics and/or trying augmentation with additional antipsychotics and antidepressants. Thompson et al found a similar rate (32.6%) of participants received adjunct psychotropic medications before their prescription of clozapine, despite the lack of robust evidence for antipsychotic polypharmacy.^29^ Although data from Thompson et al and our study is relatively old, since the National EDEN study data collection concluded in 2012, it appears that there have not been any significant advances in antipsychotic treatments for FEP in this time frame. Recent National Audit data also does not suggest that clozapine prescriptions are dramatically improving; among those eligible for clozapine, prescription rates have increased by 5% since 2017.^30^ It is also interesting to note the common prescription of olanzapine, given both the considerable side-effect burden, risk of metabolic syndrome and explicit NICE guidance on the use of olanzapine in young people under the age of 18 years, which advises that weight and body mass index monitoring is needed, but not often completed, with olanzapine.^31,32^ There is a concern that young people are being exposed to metabolic risk and being set on the path to metabolic dysfunction early in the course of psychosis, without sufficient consideration for the longer-term risks.^31^ Further, given the lack of evidence of a significantly enhanced therapeutic benefit of olanzapine in FEP,^33^ the Schizophrenia Patient Outcome Research Team do not recommend the use of olanzapine as a first-line treatment in FEP.^34^

It would be speculative to comment on the reasons for such a low clozapine-prescribing rate in EIP; however, despite their specialist psychosocial interventions, it is possible that medical management and clozapine have not featured as prominently as needed in the development of EIP services. Another potential barrier to clozapine prescribing in the UK is lack of experience or knowledge in the initiation of clozapine in the community, which may be an increased issue in areas of limited in-patient beds. In 2015, Tungaraza and Farooq conducted a survey of 243 consultant psychiatrists and identified notable knowledge deficits with regard to the efficacy, risks and benefits of clozapine; results showed that 42.7% of psychiatrists were not aware that clozapine can reduce substance use, 33% were not aware that the risk of agranulocytosis changes with time, and 20% were not aware of the benefits of clozapine in reducing risk of suicide.^35^ Furthermore, there are concerns regarding the known side-effects of clozapine, such as neutropenia and potentially fatal agranulocytosis, that are recognised to deter psychiatrists from prescribing clozapine, especially in community settings.^35,36^ Despite these reluctancies, a recent longitudinal study demonstrated that clozapine use was not associated with higher risk of severe physical morbidity; in fact, clozapine was associated with a substantially decreased mortality rate.^37^

In another survey of clinical staff conducted by Gee et al, the most commonly stated boundary to clozapine prescribing was perceived concerns regarding patient adherence to blood monitoring.^38^ Furthermore, the same authors carried out semi-structured interviews of patients eligible for treatment with clozapine and 43.4% of participants said concerns over adverse effects of clozapine were considered sufficient grounds to refuse clozapine treatment, but blood testing was not a significant barrier.^39^ In addition, 49% of participants said they would refuse clozapine if it necessitated a hospital admission.^39^ Despite these findings, it is encouraging to note the efforts in the UK within a newly established, treatment refractory service for those with schizophrenia. The Treatment Review and Assessment Team, described by Beck et al, have provided an optimistic framework for prompt clozapine initiation and management in the community, with preliminary data showing 20 patients per year are initiated on clozapine, compared with 4 community initiations before the introduction of the service.^40^

The very limited use of clozapine in the National EDEN study sample shows that barriers to clozapine prescription exist even in specialist early psychosis services, and this would be in keeping with an audit of early intervention services by the Royal College of Psychiatrists, which found that less than half of patients who were eligible for clozapine had received the drug.^41^ Yet there is evidence to suggest that earlier clozapine prescribing may have benefit in patients experiencing FEP. Lieberman et al performed a 52-week, randomised controlled trial of clozapine versus chlorpromazine in treatment-naive patients with first-episode schizophrenia and found that participants prescribed clozapine showed greater symptom improvement and earlier remission compared with participants prescribed chlorpromazine.^42^ A follow-up study by Girgis et al looked at the 9-year outcomes and found that 26.3% of participants prescribed clozapine remained on the same treatment, in contrast to 10% of those prescribed chlorpromazine.^43^ Sanz-Fuentenbro et al conducted a randomised trial of clozapine versus risperidone in treatment-naïve patients with first-episode schizophrenia, with a significant improvement in negative symptom scores in the clozapine group.^44^ Agid et al investigated response to clozapine when utilised in a standardised treatment programme in FEP; patients received two trials with two different second-generation antipsychotics, followed by a trial of clozapine as early as 25 weeks into the start of their treatment. The results were highly significant as the group prescribed clozapine demonstrated significant decreases in symptom scores compared with those who refused clozapine.^45^ Finally, a recent retrospective study of 105 treatment-resistant patients prescribed clozapine showed the length of clozapine delay (time from diagnosis of treatment resistance to initiation of clozapine) was associated with outcome, with a delay of >2.8 years having the largest effect.^46^ This is interesting as it reflects the timescales observed in the critical period for psychosis literature.^6^

One finding from our study was that several patients were chronically unwell, as demonstrated by persistently high PANSS scores, and yet were not eligible for clozapine by virtue of having only been prescribed one antipsychotic medication. This may reflect a lack of focus on the medication management of FEP or a lack of early recognition of poor prognosis. Although guidelines currently state that clozapine should be used as a third-line treatment, some authors have made a compelling argument to consider its use as a second-line treatment, in view of the fall-off in response to second-line antipsychotic therapy.^11,47^ Indeed, in a recent large-scale, three-phase trial of non-response to amisulpride in individuals with first-episode schizophrenia, Kahn et al demonstrated no added benefit to outcomes when switching to olanzapine, but concluded that greater symptomatic remission can be achieved by sequential administration of amisulpride and clozapine, providing rationale for the use of clozapine as a second-line treatment.^33^

Taking into consideration the above literature, it is clear there is a strong emerging evidence base for the use of clozapine in FEP, and moving forward, it becomes a question of how to implement an effective action plan to break down the barriers to prescribing clozapine, and ensure that eligible patients receive this efficacious treatment. Improved patient education regarding clozapine, alongside offering clozapine in the community where appropriate, may improve patient uptake in the future.^39^

### Study strengths and limitations

There are strengths to this study, including the large sample of 1027 participants enrolled in a national cohort. The National EDEN study enrolled patients from early intervention services across England over a 12-month period, making the sample highly representative.^19^ There were robust data collection techniques at baseline, 6 months and 12 months. There are, however, recognised limitations, which include the relatively small number prescribed clozapine and our working definition of ‘treatment resistance’ in FEP. Treatment adherence was not controlled for during the determination of treatment resistance, so as to not exclude participants who were potentially most unwell and further reduce our sample size. As there was a significant difference in the adherence ratings between our treatment-resistant group and the remaining EDEN sample, it is possible that some individuals who were ‘deemed’ treatment resistant may not have met this criterion had they been adherent to their medication regime.^3–5^ There was a relatively short follow-up period of 12 months; given that the literature states that the average duration of illness before initiation of clozapine is years, rather than months, this may explain the relatively small number prescribed clozapine in the large sample. Recruitment for this study concluded in 2009, with the final 12-month follow-up completed by April 2010; therefore, it is possible that there has been a shift in prescribing practices in FEP services since this time, and replication of our findings would be warranted. ICD-10 diagnoses were only available at baseline; had this data been available at follow-up, it would have given an insight into change of diagnoses over the 12-month follow-up period. Finally, this data exploration did not investigate further contextual information, such as the rates of hospital admission and relapse, or qualitative data on the barriers to prescribing clozapine in early interventions services; this would be an area for future exploration.

In conclusion, our data shows that in comprehensive national FEP services, there were significant delays in commencement of clozapine treatment for potentially eligible patients. Antipsychotic medication was often not changed despite symptom persistence, and polypharmacy was more common than use of clozapine. This may reflect a missed opportunity to influence recovery during the significant ‘critical period’. Strategies to rectify this issue may include the increased recognition of early treatment resistance as a target of therapy, including the development of definitions suitable for use in FEP services, clinical focus on the initiation of clozapine in the community, ongoing education of the benefits (including functional recovery and suicide prevention), and further emphasis on national standards for commencing clozapine in the community.^23^

## Data Availability

The corresponding and senior authors had full access to study data and had final responsibility for the decision to submit for publication. The data that support the findings of this study are available on request from the corresponding author. The data are not publicly available because of privacy or ethical restrictions.
